# Patient vs. Companion Satisfaction in the Emergency Department: Cross-sectional, Telephone-Based Survey

**DOI:** 10.5811/westjem.47351

**Published:** 2026-09-24

**Authors:** Abdo Mghames, Louisa Maria Alam, Hani Tamim, Yara Abou Harb, Eveline Hitti

**Affiliations:** * American University of Beirut Medical Center, Department of Emergency Medicine, Beirut, Lebanon; † Lebanese American University Gilbert and Rose-Marie Chagoury School of Medicine, Department of Internal Medicine, Beirut, Lebanon; ‡ American University of Beirut Medical Center, Department of Internal Medicine, Clinical Research Institute, Beirut, Lebanon; § Alfaisal University College of Medicine, Riyadh, Saudi Arabia; || American University of Beirut, Patient Affairs Office, Beirut, Lebanon

## Abstract

**Introduction:**

In Lebanon, it is common for multiple family members to accompany patients to the ED. In this cultural context, where family is closely involved with patient care, we sought to identify and compare factors associated with satisfaction among patients and their companions during the same emergency department (ED) visit.

**Methods:**

This cross-sectional, telephone-based survey compared factors associated with satisfaction among adult patients and their companions, during the same ED visit, at a tertiary-care center in Beirut, Lebanon. Adult patients presenting to the ED with intermediate- or low-acuity complaints were included in the study. We administered a questionnaire to patients and their companions covering six domains, including demographics, general admission, nursing and medical teams, discharge process, and overall satisfaction. Satisfaction was then categorized into high and low. We conducted an analysis of variables associated with high overall satisfaction for each group.

**Results:**

A total of 100 patients and 100 companions completed the survey, with a 45.7% response rate. Among companions, the top three factors associated with high overall satisfaction were: respect of confidentiality and privacy (odds ratio 12.98, 95% CI, 4.79–35.19), length of stay before being seen by triage nurse (9.77, 3.68–25.89), and overall ED length of stay (9.56, 3.7–24.37). Among patients, the top three factors associated with high overall satisfaction were: amount of time the physician spent with the patient (20.26, 7.10–57.82); overall ED length of stay (19.71, 5.44–71.39), and length of stay before being seen by the emergency physician team (13.26, 4.84–36.35). All variables were statistically significant for both patients and companions except for the discharge process, which was only significant for companions.

**Conclusion:**

This study suggests that patients and companions may have different experiences of their ED visit, highlighting the top factors associated with satisfaction for each group. Emergency department efforts aimed at enhancing patient experience should also consider processes related to companion experience in cultural contexts where family/relatives play an integral role in patient care.

## INTRODUCTION

Care of patients in the emergency department (ED) can be complex with many competing demands on resources and personnel. In addition, the unpredictability of patient flow and the diversity in case complexity present further challenges. Maintaining a consistent and positive patient experience within the dynamic environment of the ED can be difficult. Patient satisfaction is, therefore, a key quality metric for EDs and has been associated with patient outcomes including compliance with treatment plans and remission of disease.^1^,^2^

Similarly, the role of companions and family members in patient care has been shown to impact multiple health outcomes, including shorter hospital stay and faster recovery.^3^,^4^ Companions can provide emotional support, assist in decision-making, advocate for the patient’s needs, and communicate concerns.^5^ They also help the patient understand the treatment plan, often contributing significantly to overall compliance with care.^5^ While the impact of family and companions’ involvement on patient outcomes has been well established, little is known about their experience of the care journey and the variables that drive their overall satisfaction with the care received. Multiple studies have explored drivers of patient satisfaction in the ED and identified some key factors that improve patient perception of their ED care.^6^ Key among these factors is length of stay (LOS), which has consistently been found to be associated with improved patient satisfaction. Other important variables include satisfaction with the primary treating physician as well as effective communication, both of which have been shown to positively impact the patient experience.^7^,^8^

Drivers of companion satisfaction, however, have not been as well studied. Research evaluating pediatric populations in the ED found that relieving a child’s pain contributed to the satisfaction of both the child and their parent, while shorter waiting time impacted only the parent’s satisfaction.^9^ Another study identified clinical team performance and system processes as key factors associated with satisfaction for both patients and their companions. The same study, however found that the discharge process, which primarily focuses on the financial clearance of patients prior to discharge, uniquely impacted companion satisfaction.^10^ While these studies suggest that different variables may impact companion perception of the patient experience, no study to date has directly compared companion and adult patient experiences within the same visit.

In cultural contexts, where families are closely involved with patient care,^11^ understanding the companion’s encounter and drivers of their overall satisfaction, compared to that of the patient, is an important step to developing appropriate interventions to improve the experience of care in the ED. This is particularly important in Lebanon, where—unlike in the United States where most patients present to the ED alone^12^ —it is common for multiple family members to accompany patients to the ED. Our aim in this study was to identify factors associated with satisfaction among patients and their companions during the same ED visit.

## METHODS

### Study Design

We conducted a cross-sectional, telephone-based survey to compare whether adult patients and their companions presenting to the ED at a tertiary-care center in Beirut, Lebanon, perceived a different experience and level of satisfaction with ED services. We collected data between September 2022–September 2023. Confidentiality was guaranteed by removing all participant identifiers from the database after data collection. The study protocol was reviewed and approved by the institutional review board.

**Population Health Research Capsule**

What do we already know about this issue?

*The factors that drive companion satisfaction with emergency department (ED) care remain understudied compared to those influencing patient satisfaction*.

What was the research question?

*How do patient satisfaction scores compare with those of the companion who accompanies them to the ED?*

What was the major finding of the study?

*Respect for patient privacy was most valued by companions (OR 12.98, 95% CI, 4.79–35.19), while patients most valued time spent with the physician (20.26, 7.10–57.82)*.

How does this improve population health?

*Patients and companions may have different experiences in the ED, highlighting the need for a differentiated approach to improve their experiences*.

### Study Setting

Our institution is a 384-bed tertiary-care teaching hospital in Lebanon, with one of the largest EDs in the country accounting for more than 48,000 ED visits annually. The ED is staffed by a mix of emergency medicine (EM)-trained physicians and non-emergency physicians with extensive EM experience. The ED is divided into three clinical sections: high acuity, low acuity, and pediatrics. Trained nurses conducting patient assessment use the Emergency Severity Index (ESI) to categorize patients, ranging from level 1 (most critical) to level 5 (least critical).^13^ As part of the medical center’s routine patient-experience monitoring process, the Patient Affairs Office (PAO) completes phone surveys on randomly selected patients discharged from the ED within two days of discharge using an internally developed survey.

### Study Population and Study Protocol

The eligibility criteria for this study included patients aged 18 years and older who presented to the ED with either intermediate- or low-acuity complaints (ESI 3, 4, and 5) and spent a minimum of two hours in the ED. Family members were considered eligible to participate in the study if they had spent a minimum of one hour with the patient during their ED visit. The study used a stratified random sample design, with weekdays and weekends serving as the strata. By conducting data collection at various times and days of the week, the random component of this sampling was accomplished. Participants were approached and briefed about the study, emphasizing the voluntary nature of participation. If both the patient and their companion agreed to participate, they were asked to sign an informed written consent. This list was then shared with the PAO, which conducted the survey to avoid overlap with patients contacted as part of the the routine surveying process. Patients were contacted within two working days following their ED presentation and companions within five working days. Following best practices in improving survey response rates, a maximum of three phone calls were attempted within one week of the initial call.^14^ No incentives were provided for survey completion. If either companion or patient did not respond to our calls, both were excluded from the study.

Initially 219 patients and companions consented and agreed to participate in the study. Among patients 111 replied, of whom 11 were subsequently excluded because their companions failed to respond. The response rate was calculated by dividing the number of completed surveys (100) by the number of participants initially consented (219).

### Measurement

The patient satisfaction survey was developed in 2013 by ED leadership and the PAO, based on existing ED surveys published in the literature, as well as quality initiative priorities in the ED.^9^,^15^,^16^ This survey has since been used by the PAO at our institution to assess ED patient satisfaction, as part of their routine quality assurance process.^15^ The questionnaire included 30 questions and six areas of focus, including demographics, general admission, nursing and medical teams, discharge process, and overall satisfaction. The same survey was also applied to the patients’ companions for consistency. We assessed overall satisfaction, which was the primary study outcome, using a multilevel response scale. For analysis purposes, satisfaction responses were dichotomized into two groups: low satisfaction, which included responses such as “Very Poor,” “Poor,” “Fair,” and “Good” responses, and high satisfaction, which included only “Very Good” responses.

### Data Analysis

We used SPSS v28 (SPSS Statistics, IBM Corp, Armonk, NY) to clean, manage, and analyze the data. Descriptive statistics were carried out and results were reported as numbers and percentages for categorical variables, whereas we reported the mean and standard deviation for continuous variables. We calculated the association between different demographic characteristics and ED-specific information with overall satisfaction (survey question: Q20 – “Overall satisfaction with your visit to ED”), using the chi-square test for categorical variables, or the Student *t*-test for continuous variables. Results are reported as odds ratio and 95% confidence intervals. *P*-value < .05 indicated statistical significance.

## RESULTS

A total of 100 patients and 100 companions completed the survey of 219 who initially consented in each group, resulting in a 45.7% response rate. Sociodemographic characteristics between patients and companions are summarized in Table 1. Most responders were females in both groups, median age 42.59 (SD 18.09 years) for patients and 45.12 (SD 13.99 years) for companions, and most of those in both groups had an undergraduate degree; however, the results were not statistically significant. A statistically significant difference was observed for age distribution between patients and companions (*P* = .001), with companions generally being older than patients. As for marital status, there was a significant difference, with 48.0% of patients being married compared to 78.0% of companions (*P* <.001).

Tables 2 and 3 present the associations between the sociodemographic characteristics and the overall satisfaction with ED visit reported by companions and patients, respectively. Most of the companions and patients rated their overall satisfaction with their ED visit as high.

Companions who reported high satisfaction were more likely to be 30–35 years of age (40.7 vs 29.3%, *P* = .67) and accompany patients who had previously visited the ED (81.4 vs 68.3%, *P* = .13). These results were not statistically significant.

A statistically significant difference was observed in levels of satisfaction when it came to educational level, with 66.7% of those with undergraduate education reporting high satisfaction (*P* = .01), while only 9.5% of patients with graduate or postgraduate education reported high satisfaction (*P* = .01). Patients who reported high satisfaction were more likely to live in rural areas (14.3 vs 5.4%, *P* = .21), be self-payers (15.9 vs 5.4%, *P* = .20), and have previously visited the ED (81.0 vs 67.6%, *P* = .13); however, these were not statistically significant.

Figure 1 illustrates the associations (from strongest to weakest) between different elements of the companion’s experience in the ED and high overall satisfaction. All variables were statistically significant, including the discharge process. The top three factors associated with high overall satisfaction among companions were respect for confidentiality and privacy (OR 12.98, 95% CI, 4.79–35.19); LOS before being seen by triage nurse (9.77, 3.6–25.89); and overall ED LOS (9.56, 3.75–24.37).

Figure 2 illustrates the associations (from strongest to weakest) between different elements of the patient’s experience in the ED and high overall satisfaction. All variables were statistically significant except for the discharge process. The top three factors associated with high overall satisfaction among patients were the following: amount of time the physician spent with the patient (OR 20.26, 95% CI, 7.10–57.82); overall ED LOS (19.71, 5.44–71.39), and LOS before being seen by the emergency physician team (13.26, 4.84–36.35).

## DISCUSSION

Our study compared an ED patient’s experience with that of their companion regarding their overall satisfaction with ED services. A group of adult patients and their companions participated in our survey, with the majority rating their overall experience as high. Patients were generally younger than companions, and a majority of both groups had completed an undergraduate education. Patients reporting high overall satisfaction were more likely to live in rural areas, self-pay, and have visited the ED before. Companions with lower satisfaction were more likely to have presented with a patient triaged to the low-acuity section, who had not visited the ED previously. Both patients and companions considered the overall LOS in the ED as a significant factor contributing to their overall satisfaction. For patients, the top factors associated with high overall satisfaction were the amount of time the physician spent with them and their LOS before being seen by the emergency physician team. In contrast, the patients’ companions prioritized respect for confidentiality and privacy, and the LOS before seeing a triage nurse. Perceptions of discharge process uniquely influenced companion satisfaction but not patient satisfaction.

Our study was methodologically designed to understand the differences in variables associated with satisfaction for patients and companions during their ED visit, an area that has been underexplored in the literature. One prior study comparing pediatric patient satisfaction with that of their accompanying parent during the same visit found that—with the exception of pain resolution which was associated with pediatric patient satisfaction solely—factors associated with satisfaction were similar for both patients and their parents.^9^ The only other study to our knowledge that compared factors influencing adult patient experience of ED visits with those influencing the companion’s experience relied on analysis of secondary data from patient satisfaction surveys of ED visits administered to the patient (or to their companion only when the patient was unable to complete the survey for medical reasons).^10^ That approach, however, did not allow for direct comparison of satisfaction within the same ED visit. In contrast, our study is the first to examine adult patients and their companions’ experience of the same encounter, thereby providing a more accurate and methodologically robust assessment of their shared experience and potential differences in factors associated with their respective perceptions.

Our findings align with the literature, validating the impact of several key determinants on overall patient experience. While undergraduate education was associated with higher satisfaction among patients in our study, those with graduate or postgraduate education reported lower levels of satisfaction. This may reflect higher expectations among more highly educated individuals. Shorter ED LOS has been consistently associated with higher satisfaction levels across multiple studies, and our findings reinforced this association.^18^–^20^

In addition to the overall ED LOS, variables related to the treating physician, such as the amount of time the patient spent with the physician and the LOS before being seen by the physician team, were found to be significantly associated with patient satisfaction. Indeed, variables associated with the physician team have been consistently highlighted in the literature, indicating their importance in patients’ experience of services. Aspects such as overall satisfaction with the doctors^7^, doctors meeting expectations^7^, quality of patient-provider interaction^9^, satisfaction with the medical care^21^ and treatment^22^, as well as the amount of time the physician spent with the patient^21^, have all been found to be associated with patient satisfaction across the literature. In addition, nursing team variables have been linked to patient satisfaction in a few studies.^21^–^23^ While nursing-related variables were associated with patient satisfaction in our study, they did not emerge as significant factors associated with satisfaction. Furthermore, while several studies have highlighted the association of good pain control with patient satisfaction in their ED visit, this was not assessed in our study.^9^,^17^

For companions, the ED LOS also emerged as a significant factor associated with their satisfaction, consistent with Salehi et al and Natesan et al who reported that one of the most important determinants of a companion’s experiences in the ED was the length of wait times.^10^,^21^ However, our results revealed that the top drivers of companion satisfaction were respect for confidentiality and privacy, as well as the LOS before seeing a triage nurse. While privacy has been reported as a factor associated with ED patient satisfaction in other studies,^24^,^25^ this has not been previously reported as a factor associated with companion satisfaction in the existing literature. Although the LOS before triage was not strongly associated with patient satisfaction in our study, it emerged as one of the main drivers of companion satisfaction. Indeed, a study investigating factors associated with parental satisfaction revealed that their satisfaction was significantly associated with shorter waiting room times.^9^ Finally, the discharge process emerged as a unique factor associated with companion satisfaction and not patient satisfaction. One possible explanation is that companions often handle the discharge process on behalf of the patient and, thus, experience a process from which patients are generally shielded.

Our study underscores the important differences in the experiences of both the patient and their companion when evaluating and improving ED services. Our findings provide insights for policymakers and healthcare administrators when developing targeted interventions aimed at improving patient-centered care delivery and addressing the unique needs and expectations of both patients and companions within the ED setting. Enhancing workflows related to triage times, patient privacy and confidentiality, and the discharge process are key areas that warrant focused efforts to improve companions’ satisfaction. This is particularly relevant in a cultural context where companions are deeply involved in patient care, especially given reports in the literature demonstrating the positive impact of a companion in compliance to patient plans and outcomes.

## LIMITATIONS

This study has some limitations. First, the relatively small sample size constrained the statistical power of our analyses and contributed to the wide confidence intervals observed in some of the results. Second, the single-center design and limited response rate may restrict the generalizability of our results. Third, the cross-sectional design of this study did not enable causality to be established. In addition, the data was gathered through self-reporting by patients and companions, which may have introduced recall bias. Finally, we acknowledge the potential for selection bias due to non-response. Demographic data on non-responders was not collected for analysis.

## CONCLUSION

This study suggests that patients and their companions may have different perceptions of their ED stay, with patients valuing time spent with the treating physician of utmost importance, while companion satisfaction was tied to respect for privacy and confidentiality. Efforts aimed at enhancing patient experience in the ED should consider processes related to companion experience, especially in cultural contexts where family plays an integral role in patient care.

## Figures and Tables

**Figure 1:**
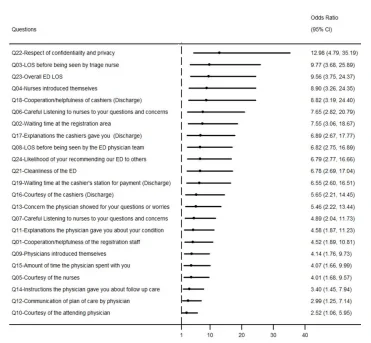
Association between elements of companion satisfaction and high overall satisfaction in a cross-sectional study assessing emergency department satisfaction. *ED*, emergency department; *LOS*, length of stay; *OR*, odds ratio

**Figure 2:**
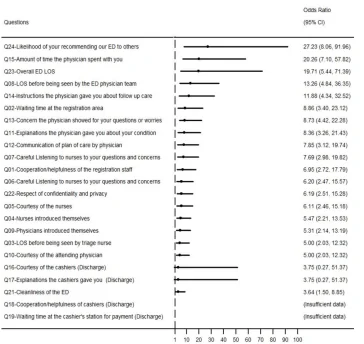
Association between different elements of patient satisfaction and high overall satisfaction in a cross-sectional study assessing emergency department satisfaction. *ED*, emergency department; *LOS*, length of stay; *OR*, odds ratio.

**Table 1:** Differences in sociodemographic characteristics between patients and their companions in a cross-sectional study assessing emergency department satisfaction.

Variables	Patients (N = 100)	Companions (N = 100)	P-value
Sex (male)	39 (39.0%)	48 (48.0%)	.20
Age (mean, SD)	42.59 (18.09)	45.12 (13.99)	.27
[18–30]	36 (36.0%)	18 (18.0%)	.001
[30–45]	26 (26.0%)	36 (36.0%)	
[45–65]	21 (21.0%)	38 (38.0%)	
Marital Status			
Married	48 (48.0%)	78 (78.0%)	< .001
Educational level			
Up to high school	24 (24.0%)	19 (19.0%)	55
Undergraduate	58 (58.0%)	58 (58.0%)	
Graduate/postgraduate	18 (18.0%)	23 (23.0%)	
Living location			
Rural	11 (11.0%)	17 (17.0%)	.22

**Table 2:** Associations between sociodemographic characteristics and high overall satisfaction among companions (N = 100) in a cross-sectional study assessing emergency department satisfaction.

Variables	Low satisfaction (n = 41, 41.0%)	High satisfaction (n = 59, 59.0%)	P-value	OR (95% CI)
Sex				
Male	22 (53.7%)	26 (44.1%)	.35	0.68 (0.31, 1.52)
Age (mean, SD)	45.41 (14.24)	44.92 (13.93)	.73	1.00 (0.97, 1.03)
[18–30]	8 (19.5%)	10 (17.0%)		Reference
[30–45]	12 (29.3%)	24 (40.7%)	.67	1.60 (0.50, 5.10)
[45–65]	18 (43.9%)	20 (33.9%)		0.89 (0.29, 2.74)
>65	3 (7.3%)	5 (8.5%)		1.33 (0.24, 7.35)
Marital Status				
Married	32 (78.1%)	46 (78.0%)	.99	1.00 (0.38, 2.60)
Educational level				
Up to high school	6 (14.6%)	13 (22.0%)		Reference
Undergraduate	26 (63.4%)	32 (54.2%)	.58	0.57 (0.19, 1.70)
Graduate/ postgraduate	9 (22.0%)	14 (23.7%)		0.72 (0.20, 2.58)
Living location				
Rural	8 (19.5%)	9 (15.3%)	.58	0.74 (0.26, 2.12)
Guarantor type				
Self-pay	5 (12.2%)	7 (11.9%)	1.00	0.97 (0.29, 3.30)
ED				
Low Acuity	21 (51.2%)	22 (37.3%)	.17	0.57 (0.25, 1.27)
Patient’s first visit to ED				
No	28 (68.3%)	48 (81.4%)	.13	2.03 (0.80, 5.13)
On shift*				
Off shift	29 (70.7%)	43 (72.9%)	.81	1.11 (0.46, 2.69)

* “On shift” refers to weekdays and mornings, whereas “Off shift” includes evenings, nights, and weekends.

*OR*, odds ratio.

**Table 3:** Associations between sociodemographic characteristics and high overall satisfaction among patients (N = 100), in a cross-sectional study assessing emergency department (ED) satisfaction.

Variables	Low (n = 37, 37.0%)	High (n = 63, 63.0%)	P-value	OR (95% CI)
Sex				
Male	14 (37.8%)	25 (39.7%)	.86	1.08 (0.47, 2.49)
Age (mean, SD)	37.73 (14.74)	45.44 ± 19.35	.10	1.03 (1.00, 1.05)
[18–30]	14 (37.8%)	22 (34.9%)		Reference
[30–45]	13 (35.1%)	13 (20.6%)	.19	0.64 (0.23, 1.76)
[45–65]	7 (18.9%)	14 (22.2%)		1.27 (0.41, 3.93)
>65	3 (8.1%)	14 (22.2%)		2.97 (0.72, 12.23)
Marital Status				
Married	17 (46.0%)	31 (49.2%)	.75	1.14 (0.51, 2.57)
Educational level				
Up to high school	9 (24.3%)	15 (23.8%)		Reference
Undergraduate	16 (43.2%)	42 (66.7%)	.01	1.57 (0.58, 4.31)
Graduate/ postgraduate	12 (32.4%)	6 (9.5%)		0.30 (0.08, 1.08)
Living location				
Rural	2 (5.4%)	9 (14.3%)	.21	2.92 (0.59, 14.30)
Guarantor type				
Self-pay	2 (5.4%)	10 (15.9%)	.20	3.30 (0.68, 15.98)
ED				
Low Acuity	16 (43.2%)	27 (42.9%)	.97	0.98 (0.43, 2.23)
Patient’s first visit to ED				
No	25 (67.6%)	51 (81.0%)	.13	2.04 (0.80, 5.18)
On shift*				
Off shift	27 (73.0%)	45 (71.4%)	P .87	0.93 (0.37, 2.30)

* “On shift” refers to weekdays and mornings, whereas “Off shift” includes evenings, nights, and weekends.

*OR*, odds ratio.

## References

[1] Renzi C, Abeni D, Picardi A (2001). Factors associated with patient satisfaction with care among dermatological outpatients. Br J Dermatol.

[2] Hurwitz EL, Morgenstern H, Yu F (2005). Satisfaction as a predictor of clinical outcomes among chiropractic and medical patients enrolled in the UCLA low back pain study. Spine (Phila Pa 1976).

[3] Mackie BR, Mitchell M, Marshall A (2018). The impact of interventions that promote family involvement in care on adult acute-care wards: An integrative review. Collegian.

[4] Giap TT, Park M (2021). Implementing patient and family involvement interventions for promoting patient safety: a systematic review and meta-analysis. J Patient Saf.

[5] Ellingson LL (2002). The roles of companions in geriatric patient–interdisciplinary oncology team interactions. J Aging Stud.

[6] Aleksandrovskiy I, Ganti L, Simmons S (2022). The Emergency Department patient experience: In their own words. J Patient Exp.

[7] Abidova A, Alcântara da Silva P, Moreira S (2020). Predictors of patient satisfaction and the perceived quality of healthcare in an emergency department in Portugal. West J Emerg Med.

[8] Abu Ammo M, Abu-Shaheen AK, Kobrosly S (2014). Determinants of patient satisfaction at tertiary care centers in Lebanon. Open J Nurs.

[9] Magaret ND, Clark TA, Warden CR (2002). Patient satisfaction in the emergency department--a survey of pediatric patients and their parents. Acad Emerg Med.

[10] Natesan P, Hadid D, Harb YA (2019). Comparing patients and families perceptions of satisfaction and predictors of overall satisfaction in the emergency department. PLoS One.

[11] Natesan P, Hadid D, Abou Harb Y (2019). Comparing patients and families perceptions of satisfaction and predictors of overall satisfaction in the emergency department. PLoS One.

[12] Totten VY, Bryant TK, Chandar AK (2014). Perspectives on visitors in the emergency department: their role and importance. J Emerg Med.

[13] Tanabe P, Travers D, Gilboy N (2005). Refining Emergency Severity Index triage criteria. Acad Emerg Med.

[14] Phillips AW, Friedman BT, Durning SJ (2017). How to calculate a survey response rate: best practices. Acad Med.

[15] Yarris LM, Frakes B, Magaret N (2012). How accurately can emergency department providers estimate patient satisfaction?. West J Emerg Med.

[16] Soleimanpour H, Gholipouri C, Salarilak S (2011). Emergency department patient satisfaction survey in Imam Reza Hospital, Tabriz, Iran. Int J Emerg Med.

[17] Fontova-Almató A, Suñer-Soler R, Juvinyà-Canal D (2019). Factors associated with patients’ and companions’ satisfaction with a hospital emergency department: a descriptive, cross-sectional study. Nurs Open.

[18] Viotti S, Cortese CG, Garlasco J (2020). The buffering effect of humanity of care in the relationship between patient satisfaction and waiting time: a cross-sectional study in an emergency department. Int J Environ Res Public Health.

[19] Reznek MA, Larkin CM, Scheulen JJ (2021). Operational factors associated with emergency department patient satisfaction: analysis of the Academy of Administrators of Emergency Medicine/Association of Academic Chairs of Emergency Medicine national survey. Acad Emerg Med.

[20] de Steenwinkel M, Haagsma JA, van Berkel ECM (2022). Patient satisfaction, needs, and preferences concerning information dispensation at the emergency department: a cross-sectional observational study. Int J Emerg Med.

[21] Salehi T, Nayeri ND, Mohammadi E (2020). Exploring patients and family members’ experiences of care in the emergency department. Emerg Nurse.

[22] Reihani H, Pishbin E, Abbasi Shaye Z Patient satisfaction analysis in emergency department in Imam Reza Hospital of Mashhad. J Patient Saf Qual Improv.

[23] Reihani H, Pishbin E, Abbasi Shaye Z (2015). Patient satisfaction analysis in emergency department in Imam Reza Hospital of Mashhad. Patient Safety Qual Improv J.

[24] Nayeri ND, Aghajani M (2010). Patients’ privacy and satisfaction in the emergency department: a descriptive analytical study. Nurs Ethics.

[25] Lin YK, Lin CJ (2011). Factors predicting patients’ perception of privacy and satisfaction for emergency care. Emerg Med J.

